# *In Vivo* Corneal Biomechanical Properties in a Selected Chinese Population, Measured Using the Corneal Visualization Scheimpflug Technology

**DOI:** 10.3389/fbioe.2022.863240

**Published:** 2022-04-13

**Authors:** Yuxin Li, Lei Tian, Li-Li, Guo, Yiran Hao, Ying Jie

**Affiliations:** ^1^ Beijing Ophthalmology and Visual Sciences Key Laboratory, Beijing Tongren Eye Center, Beijing Tongren Hospital, Beijing Institute of Ophthalmology, Capital Medical University, Beijing, China; ^2^ Beijing Chaoyang Hospital, Capital Medical University, Beijing, China; ^3^ Beijing Advanced Innovation Center for Big Data-Based Precision Medicine, Beihang University, Capital Medical University, Beijing, China; ^4^ The First People’s Hospital of Xuzhou, Xuzhou, China

**Keywords:** CorVis ST, repeatability, reproducibility, intraclass correlation coefficient, correlation

## Abstract

**Purpose:** To evaluate the repeatability and reproducibility of recalculated dynamic corneal response (DCR) parameters and the biomechanical-compensated intraocular pressure (bIOP) derived from the Corneal Visualization Scheimpflug Technology (Corvis ST), as well as to study the variations of DCR parameters and their relationship with demographic, and ocular characteristics.

**Methods:** A total of 544 healthy subjects were recruited in this study and a series of ophthalmological examinations were performed on their right eyes. Three repeated measurements were obtained at 3-min intervals for 291 of the participants to ensure repeatability. A sum of 100 participants was examined twice within 2-h intervals using two different Corvis ST in the reproducibility study. The repeatability and reproducibility of 37 parameters, including 36 DCR parameters and bIOP, were assessed by the coefficient of repeatability (CR), coefficient of variation (CV), intraclass correlation coefficient (ICC), and within-subject standard deviation (sw). Pearson’s correlation coefficients and stepwise multivariate linear regression models were performed to investigate whether the DCR parameters were related to demographic and ocular characteristics.

**Results:** Of all the 37 parameters, 34 showed excellent (ICC ≥0.90) or good (ICC ≥0.75) repeatability while 27 of the 37 parameters showed excellent (ICC ≥0.90) or good (ICC ≥0.75) reproducibility. In particular, a CV of less than 20% was found for all DCR parameters and bIOP. A fraction of 14 out of 36 DCR parameters was selected for correlation analysis, based on measurement reliability and clinical relevance in referring to previous literature. Age was negatively associated with the Highest concavity delta arc length (HCdArcL) and peak distance (PD) but it positively correlated with the Whole Eye Movement Max Length (WEMML). Intraocular pressure (IOP) and central corneal thickness (CCT) were negatively associated with the deformation amplitude ratio (DARM) [1 mm], A1 Velocity (A1V), and PD, while positively related to the stiffness parameter at applanation 1 (SP-A1). The bIOP was negatively associated with A1V but positively associated with A2 Velocity (A2V). The anterior chamber volume (ACV) was negatively associated with the pachy slope (PS), WEMML, and SP-A1.

**Conclusion.** The Corvis ST showed good precision for the repeatability and reproducibility of 36 DCR parameters and bIOP parameters in healthy eyes. The IOP, CCT, bIOP, Km, and ACV significantly influenced the DCR parameters of the eyes.

## Introduction

The investigation of corneal biomechanics recently gained space as a hot topic in ophthalmology mainly due to its wide applications (Roberts etal., 2014). Corneal biomechanical properties were known to assist with detecting corneal diseases (Vinciguerra et al., 2017a; Reinprayoon et al., 2021; Ziaei et al., 2021; Henriquez et al., 2022), predicting refractive outcomes before corneal refractive surgery (Chen et al., 2018), judging different protocols of collagen crosslinking treatments (Greenstein et al., 2012), and even selecting intracorneal ring implants (Bao et al., 2016; Shen et al., 2022).

Multifarious methods have been devised to study the biomechanics of cornea *in vivo* (Hollman et al., 2013; Flockerzi et al., 2021). The Ocular Response Analyzer (ORA; Reichert, Buffalo, NY, United States) which provided great knowledge of the corneal biomechanics was one of the methods that were used in the clinic (Luce, 2005) but it cannot exhibit the dynamic corneal response (DCR) parameters. In 2010, a visual display method that was based on corneal dynamic deformation video using an ultra-fast Scheimpflug camera combined with a classic non-contact tonometer was introduced (Eliasy et al., 2018; Krysik et al., 2018; Guo et al., 2021). The non-contact tonometer is called corneal visualization Scheimpflug technology (Corvis ST; Oculus Optikgeräte GmbH, Wetzlar, Germany). The dependence of available parameters on specific disease entities was investigated, together with the changes in their values after performing surgical procedures. Furthermore, in terms of evaluating the impact of biomechanical parameters, the software versions have been changed several times with new parameters. The newer Corvis ST version (1.6r2042) includes the biomechanically corrected IOP (bIOP) (Joda et al., 2015; Salouti et al., 2022) and DCR parameters like max inverse radius (MIR), deformation amplitude ratio (DARM) [1 mm], deformation amplitude ratio (DARM) [2 mm], pachy slope (PS), Ambrosio relational thickness to the horizontal profile (ARTh), integrated radius (IR), stiffness parameter at applanation 1 (SP-A1) (Jędzierowska and Koprowski, 2019), and Corvis Biomechanical Index (CBI) (Vinciguerra et al., 2016a; Vinciguerra et al., 2017a; Vinciguerra et al., 2017b). For diagnosis and follow-up purposes, the reliability of these measurements is important, and its evaluation is carried out by analyzing repeatability and reproducibility factors. Additionally, independent studies should focus on determining “normal” values for different populations so that new technologies can establish wider acceptance and broad utility at clinical levels.

Therefore, this study aimed to evaluate the repeatability and reproducibility of Corvis ST parameters. Moreover, we also demonstrated variations in biomechanical properties that are provided by Corvis ST in healthy Chinese participants, in addition to their relationship with demographic and ocular characteristics.

## Methods

### Subject Recruitment

A total of 544 healthy Chinese participants aged between 10 and 75 years were recruited at Beijing TongRen Hospital, which is affiliated to Capital Medical University. The recruitment was done between January 2021 and January 2022. This cross-sectional study was approved by the office of Research Ethics Committee of Beijing TongRen Hospital, in accordance with the principles of the Helsinki Declaration. All the participants provided informed consents before taking part in the study.

### Ocular Examinations

All participants underwent a complete ophthalmic examination and a standardized interview procedure. Ophthalmic examination included detailed visual acuity assessment; slit-lamp microscopy and fundus examination; corneal and anterior chamber tomography with Pentacam (Oculus Optikgeräte GmbH, Wetzlar, and Germany); as well as corneal biomechanics and intraocular pressure with Corvis ST. To reduce the effect of diurnal variation, all assessments were performed on a single visit.

The study excluded participants if they had previous corneal or ocular surgery, any ocular pathology or systemic disease that affects the eye, or long-term use of topical ocular medications.

### Corvis ST Measurement

IOP and corneal biomechanical parameters were measured by Corvis ST, a noncontact tonometer and imaging device that can provide additional information on the corneal response to specific airflow pulses. An ultrahigh-speed Scheimpflug camera (recorded at 4,330 frames per second) captured corneal deformations in the horizontal range of 8.5 mm. A video clip containing 140 digital frames corresponded to a recording time of 33 msec. The details of measurements on the Corvis ST are described elsewhere (Robert et al., 2019; Zhang et al., 2021a). In the latest release of the software, more DCR parameters [MIR, DARM (1 mm), DARM (2 mm) PS, ARtH, IR, SP-A1, and CBI) were introduced, together with bIOP. (Hirasawa et al., 2018).

Table 1 shows the abbreviations and interpretations of the 36 DCR parameters and bIOP parameter that were measured by the Corvis ST. The latest version of the Oculus software (version 1.6r2042) was used to recalculate all the Corvis ST measurements and this facilitated more precise parameters and data association. The quality specification section on the output graph was used to check the quality. An “OK” reading was interpreted to reflect an acceptable quality.

**TABLE 1:** Abbreviations and explanations of parameters measured by Corvis ST.

Abbreviations of parameter	Full name	Explanation
DAM (mm)	Deformation amplitude Max	Max length at deformation amplitude
A1T (ms)	First applanation time	Time from the initiation of the air puff until the first applanation
A2T (ms)	Second applanation time	Time from the initiation of the air puff until the second applanation
HCT (ms)	Highest concavity time	Time from the start until highest concavity of the cornea is reached
A1V (m/s)	A1 Velocity	Corneal velocity at the first applanation
A2V (m/s)	A2 Velocity	Corneal velocity at the second applanation
PD (mm)	Peak distance	Distance of the two surrounding “knees” at highest concavit
Radius (mm)	Radius	Radius of curvature at the time of highest concavity
A1DA (mm)	A1 Deformation Amplitude	Deformationtion amplitude at the first applanation
HCDA (mm)	Highest concavity Deformation Amplitude	Deformation amplitude at the maximum deformation
A2DA (mm)	A2 Deformation Amplitude	Deformation amplitude at the second applanation
A1DLL (mm)	A1 Deflection Length	Deflection length at the first applanation
A2DLL (mm)	A2 Deflection Length	Deflection length at second applanation
HCDLL (mm)	Highest concavity Deflection Length	Deflection length at maximum deformation
A1DLA (mm)	A1 Deflection Amplitude	Deflection Amplitude deflection amplitude at the first applanation
A2DLA (mm)	A2 Deflection Amplitude	Deflection Amplitude deflection amplitude at the second applanation
HCDLA (mm)	Highest concavity Deflection Amplitude	Deflection Amplitude deflection amplitude at the maximum deformation
DLAML (mm)	Deflection Amplitude Max Length	Max Length at deflection amplitude
DLAMT (ms)	Deflection Amplitude Max Time	Max time at deflection amplitude
WEMML (mm)	Whole Eye Movement Max Length	Max length of whole eye movement
WEMMT (ms)	Whole Eye Movement Max Time	Max time of whole eye movement
A1DLAr (mm)	A1 Deflection Area	Deflection area at the first applanation
A2DLAr (mm)	A2 Deflection Area	Deflection area at the second applanation
HCDLAr (mm)	Highest concavity Deflection Area	Deflection area at the maximum deformation
A1dArcL (mm)	A1 dArc Length	Delta arc length at the first applanation
A2dArcL (mm)	A2 dArc Length	Delta arc length at the second applanation
HCdArcL (mm)	Highest concavity dArc	Length Delta arc length at the maximum deformation
dArcLM (mm)	dArcLengthMax	Max delta arc length
MIR (mm^−1^)	Max Inverse Radius	The maximum value of radius of curvature during concave phase of the deformation
PS (μm)	Pachy Slope	Pachy Slope
IR (mm^−1^)	Integrated Radius	Integrated Radius
DARM [1 mm]	Deformation amplitude ratio [1 mm]	The maximum value of the ratio between deformation amplitude at the apex 1 mm from central cornea
DARM [2 mm]	Deformation amplitude ratio [2 mm]	The maximum value of the ratio between deformation amplitude at the apex 2 mm from central cornea
ARTh	Ambrosio relational thickness to the horizontal profile	Ambrosio relational thickness to the horizontal profile
bIOP (mmHg)	Biomechanical Intra Occular Pressure	Biomechanical Intra Occular Pressure
SP-A1	Stiffness parameter at applanation 1	The adjusted pressure at A1 (adjusted AP1) minus a biomechanically corrected IOP value (bIOP) and then divided by A1 deflection amplitude, stiffness parameter at first applanation
CBI	Corvis biomechanical index	Corvis biomechanical index

A total of 291 participants received three measurements which were repeated until all parameters were obtained with acceptable quality. This was done to determine the intra-observer repeatability. Between measurements, the cornea was allowed to recover from the air puff by resting for 3 minutes. To assess the inter-device reproducibility, a subgroup of 100 patients was randomly selected and analyzed separately. The measurements were performed using two different devices, although they had the same software (version 1.6r2042). The second batch of measurements was taken 2 hours after those of the first batch were taken.

### Statistical Analysis

The SPSS version 26.0 (SPSS, Inc., Chicago, IL, United States) was used for statistical analysis. The normality of distribution of the measured variables was estimated using the Kolmogorov-Smirnov test. The recalculated biomechanical parameters were analyzed for repeatability and reproducibility. The statistical significance of the coefficient of repeatability (CR), coefficient of variation (CV), intraclass correlation coefficient (ICC), and within-subject standard deviation (sw) were used to evaluate the parametric repeatability analysis (Herber et al., 2020). The CV values that were less than 20% were considered to reflect high repeatability (Ali et al., 2014). The explanation of the ICC was based on the following stipulations: >0.90 means excellent repeatability, 0.75–0.90 correlates to good repeatability, and <0.75 reflects poor to moderate repeatability of clinical measurement (Ali et al., 2014).

Based on the reliability of the measurements, as well as the clinical relevance with reference to previous literature, 14 of the 36 DCR parameters were selected for correlation analysis (Vinciguerra et al., 2016b; Cui et al., 2019). Pearson’s correlation analysis was used to explore the relationship between the DCR parameters and demographic/ocular characteristics, including age, IOP, CCT, bIOP, mean keratometry (Km), and anterior chamber volume (ACV). Stepwise multivariate linear regression analysis was carried out, where all significantly-changed parameters that were retrieved from Pearson’s correlation analysis were regarded as the independent variables while the demographic/ocular characteristics were dependent variables. A *p-*value < 0.05 was interpreted as statistically significant.

## Results

### Characteristics of Healthy Participants

A total of 544 healthy Chinese participants were recruited in this study. Table 2 shows the demographic data of all the participants. The mean values of the DCR parameters in the eyes, together with the corresponding SD and range are shown in Table 3.

**TABLE 2:** Demographic and ocular data of participants.

Parameters	All (n = 544)
Age (y)	29.1 ± 12.6 (10–75)
Gender (n)	280 females, 264 males
IOP (mmHg)	14.82 ± 2.92 (8.0–25.5)
bIOP (mmHg)	14.64 ± 2.61 (6.8–23.1)
CCT (μm)	545.9 ± 30.1 (389–638)
Km (D)	43.33 ± 1.38 (36.8–47.1)
Astig (D)	1.20 ± 0.73 (0–3.0)
ACV (mm^3^)	183.4 ± 45.3 (53–280)

IOP, intraocular pressure, bIOP, biomechanical-compensated intraoocular pressure, CCT, Central corneal thickness, Astig, astigmia, Km, mean keratometry, ACV, nterior chamber volume.

**TABLE 3:** Dynamic Corneal Response parameters data of participants.

Parameters	Mean	±SD	Range
DAM (mm)	1.03	0.10	0.76–1.45
A1T (ms)	7.44	0.33	6.72–8.75
A1V (ms)	0.13	0.02	0.07–0.18
A2T (ms)	21.76	0.37	20.60–23.21
A2V (ms)	−0.27	0.04	−0.42–0.16
HCT (ms)	16.91	0.47	15.54–18.45
PD (mm)	5.01	0.31	3.93–6.12
R (mm)	7.09	0.76	5.26–10.11
A1DA (mm)	0.13	0.01	0.10–0.17
HCDA (mm)	1.03	0.10	0.76–1.45
A2DA (mm)	0.36	0.07	0.07–0.68
A1DLL (mm)	2.27	0.19	0.40–2.85
HCDLL (mm)	6.54	0.49	4.86–8.23
A2DLL (mm)	2.77	0.57	1.39–5.60
A1DLA (mm)	0.09	0.01	0.04–0.14
HCDLA (mm)	0.89	0.11	0.56–1.32
A2DLA (mm)	0.11	0.08	0.05–1.85
DLAM (mm)	0.90	0.12	0.59–2.03
DLAM (ms)	16.19	0.78	4.00–20.62
WEMML (mm)	0.26	0.07	0.12–0.55
WEMMT (ms)	21.46	0.43	19.75–23.81
A1DLAR (mm^2^)	0.17	0.03	0.10–0.26
HCDLAR (mm^2^)	3.22	0.56	1.69–5.59
A2DLAR (mm^2^)	0.24	0.25	0.03–5.88
A1DARCL (mm)	−0.02	0.01	−0.03–0.06
HCDARCL (mm)	−0.13	0.02	−0.23–0.01
A2DARCL (mm)	−0.02	0.02	−0.45–0.06
DARCLM (mm)	−0.15	0.03	−0.49–0.02
MIR (mm^−1^)	0.17	0.02	0.13–0.26
DARM (2 mm)	4.19	0.33	3.29–5.36
PS (μm)	48.25	9.27	1.62–76.94
DARM [1 mm]	1.58	0.05	1.43–1.80
ARTH	431.16	84.58	273.18–990.06
IR (mm^−1^)	8.09	0.93	5.42–10.71
SP-A1	103.53	17.52	59.49–163.20
CBI	0.11	0.13	0.00–0.44

### Repeatability and Reproducibility

Table 4 showed the repeatability and reproducibility values of the Corvis ST parameters. Among the 37 parameters, 20 (54.05%) had excellent repeatability (ICC ≥0.90), 14 (37.84%) had good repeatability (ICC ≥0.75), and 3 (8.11%) had poor to moderate repeatability. The CV of all the DCR parameters was less than 20%. Particularly, 12 of 37 parameters (32.43%) were highly reliable (CV < 5%). All the new parameters showed good or excellent repeatability.

**TABLE 4:** Repeatability and reproducibility of the Corvis ST parameters in healthy eyes.

Parameter	Repeatability				Reproducibility			
	sw	CR	CV (%)	ICC(95%CI)	sw	CR	CV (%)	ICC(95%CI)
DAM (mm)	0.05	0.14	4.75	0.93 (0.91–0.94)	0.06	0.15	5.20	0.88 (0.82–0.92)
A1T (ms)	0.17	0.46	2.32	0.92 (0.91–0.94)	0.18	0.50	2.50	0.88 (0.82–0.92)
A1V (m/s)	0.01	0.02	6.03	0.92 (0.90–0.93)	0.01	0.03	6.80	0.87 (0.80–0.91)
A2T (ms)	0.16	0.45	0.74	0.94 (0.93–0.95)	0.18	0.51	0.84	0.90 (0.86–0.94)
A2V (m/s)	0.02	0.05	-6.12	0.92 (0.90–0.93)	0.02	-12.63	-6.45	0.87 (0.81–0.91)
HCT (ms)	0.37	1.01	2.16	0.65 (0.58–0.72)	0.38	1.06	2.26	0.53 (0.29–0.68)
PD (mm)	0.15	0.41	2.89	0.93 (0.91–0.94)	0.16	0.44	3.10	0.88 (0.82–0.92)
Radius (mm)	0.54	1.49	7.97	0.81 (0.77–0.85)	0.56	1.54	8.32	0.68 (0.53–0.79)
A1DA (mm)	0.01	0.02	5.70	0.84 (0.80–0.87)	0.01	0.02	6.13	0.75 (0.63–0.83)
HCDA (mm)	0.05	0.14	4.55	0.94 (0.92–0.95)	0.05	0.14	4.86	0.88 (0.82–0.92)
A2DA (mm)	0.03	0.08	8.74	0.93 (0.91–0.94)	0.03	0.10	9.71	0.87 (0.80–0.91)
A1DLL (mm)	0.12	0.34	5.55	0.83 (0.80–0.86)	0.13	0.35	5.79	0.76 (0.64–0.84)
HCDLL (mm)	0.22	0.61	3.33	0.92 (0.91–0.94)	0.24	0.66	3.63	0.87 (0.81–0.91)
A2DLL (mm)	0.38	1.04	14.60	0.82 (0.78–0.85)	0.38	1.07	15.04	0.68 (0.53–0.79)
A1DLA (mm)	0.01	0.02	6.54	0.84 (0.81–0.87)	0.01	0.02	7.60	0.69 (0.50–0.79)
HCDLA (mm)	0.04	0.12	4.68	0.96 (0.95–0.96)	0.05	0.14	5.32	0.91 (0.86–0.94)
A2DLA (mm)	0.01	0.03	9.51	0.86 (0.84–0.89)	0.01	0.03	9.95	0.67 (0.50–0.78)
DLAML (mm)	0.04	0.12	4.60	0.95 (0.94–0.96)	0.05	0.13	5.04	0.91 (0.86–0.94)
DLAMT (ms)	0.43	1.19	2.64	0.56 (0.47–0.64)	0.49	1.34	3.00	0.31 (0.03–0.53)
WEMML (mm)	0.03	0.09	12.02	0.92 (0.91–0.94)	0.03	0.09	12.23	0.88 (0.82–0.92)
WEMMT (ms)	0.26	0.71	1.19	0.86 (0.83–0.89)	0.28	0.78	1.31	0.75 (0.63–0.83)
A1DLAr (mm^2^)	0.02	0.06	14.22	0.74 (0.74–0.82)	0.03	0.08	14.80	0.65 (0.47–0.76)
HCDLAr (mm^2^)	0.22	0.60	6.37	0.96 (0.95–0.96)	0.22	0.62	6.54	0.93 (0.89–0.95)
A2DLAr (mm^2^)	0.04	0.10	17.44	0.75 (0.75–0.83)	0.03	0.09	16.51	0.60 (0.41–0.73)
A1dArcL (mm)	0.00	0.01	−14.26	0.85 (0.81–0.88)	0.00	0.01	−15.57	0.79 (0.55–0.79)
HCdArcL (mm)	0.02	0.06	−18.12	0.85 (0.82–0.88)	0.02	0.06	−18.32	0.68 (0.53–0.79)
A2dArcL (mm)	0.00	0.01	−18.17	0.83 (0.79–0.86)	0.00	0.01	−18.33	0.82 (0.73–0.88)
dArcLM (mm)	0.02	0.06	−14.17	0.90 (0.88–0.92)	0.02	0.06	−14.38	0.83 (0.74–0.88)
New parameters								
MIR (mm^−1^)	0.01	0.04	7.37	0.87 (0.84–0.89)	0.01	0.04	7.72	0.74 (0.61–0.82)
DARM [2 mm]	0.36	0.10	7.70	0.89 (0.86–0.91)	0.36	1.00	7.84	0.77 (0.66–0.85)
PS (μm)	5.78	16.00	9.84	0.98 (0.97–0.98)	5.12	14.18	9.07	0.94 (0.91–0.96)
DARM [1 mm]	0.00	0.11	2.36	0.88 (0.85–0.90)	0.04	0.11	2.55	0.75 (0.63–0.83)
ARTh	26.31	72.87	6.62	0.99 (0.98–0.99)	26.77	74.16	6.69	0.98 (0.97–0.99)
bIOP (mmHg)	0.85	2.34	6.35	0.96 (0.95–0.97)	0.95	2.64	6.92	0.95 (0.92–0.96)
IR (mm^−1^)	0.47	1.29	5.27	0.96 (0.96–0.97)	0.52	1.44	5.90	0.91 (0.87–0.94)
SP-A1	6.21	17.20	7.29	0.97 (0.97–0.98)	6.50	18.01	7.66	0.95 (0.92–0.97)
CBI	0.13	0.37	17.69	0.91 (0.89–0.93)	0.13	0.37	18.54	0.82 (0.73–0.88)

CV, coefficient of variation; ICC, interclass correlation coefficient; CI, Confidence interval; RC, repeatability coefficient; Sw, within-subject SD.

Out of the 37 parameters, 9 (24.32%) showed excellent reproducibility (ICC ≥0.90), 18 (48.65%) showed good (ICC ≥0.75), and 10 (27.03%) showed poor to moderate repeatability (ICC d<0.75). A CoV value that was less than 20% was found for each of the DCR parameters. A fraction of 9 out of 37 parameters (24.32%) were highly reliable (CV <5%). All the other new parameters showed good or excellent reproducibility, except for MIR.

### Determinants of Dynamic Corneal Response Parameters

Table 5 shows the results of the Pearson’s correlation analysis. All the parameters that statistically correlated with age, IOP, CCT, bIOP, Km, Astig, and ACV were selected in a linear regression model using stepwise selection.

**TABLE 5:** Correlation of demographics/ocular characteristics and corneal biomechanical parameters.

Variables	Age(y)		IOP(mmHg)		CCT (μm)		bIOP(mmHg)		Km(D)		Astig(D)		ACV	
	rho	P	rho	P	Rho	P	rho	P	rho	P	Rho	P	rho	P
A1V (m/s)	0.106	0.013	−0.806	0.000	−0.177	0.000	−0.74	0.000	0.038	0.376	0.007	0.867	0.078	0.070
A2V (m/s)	0.003	0.948	0.667	0.000	0.064	0.14	0.643	0.000	0.017	0.691	−0.044	0.309	−0.134	0.002
PD (mm)	−0.093	0.030	−0.707	0.000	−0.140	0.001	−0.629	0.000	−0.364	0.000	0.003	0.951	0.319	0.000
Radius (mm)	0.065	0.130	0.255	0.000	0.211	0.000	0.179	0.000	−0.120	0.005	−0.187	0.000	−0.079	0.065
HCDLA (mm)	−0.038	0.377	−0.776	0.000	−0.159	0.000	−0.702	0.000	−0.155	0.000	0.033	0.447	0.279	0.000
WEMML (mm)	0.341	0.000	−0.034	0.432	0.018	0.675	-0.101	0.019	0.182	0.000	−0.148	0.001	−0.418	0.000
HCdArcL (mm)	−0.099	0.022	0.294	0.000	−0.166	0.000	0.357	0.000	−0.054	0.208	0.124	0.004	−0.007	0.876
DARM [1 mm]	−0.081	0.058	−0.524	0.000	−0.468	0.000	−0.357	0.000	0.161	0.000	0.061	0.155	0.217	0.000
DARM [2 mm]	0.001	0.978	−0.564	0.000	−0.464	0.000	−0.407	0.000	0.209	0.000	0.014	0.742	0.216	0.000
PS (μm)	−0.038	0.373	0.141	0.001	0.248	0.000	0.065	0.128	0.166	0.000	0.104	0.015	−0.348	0.000
ARTh	0.022	0.615	−0.049	0.251	0.144	0.001	−0.103	0.017	−0.161	0.000	−0.051	0.237	0.192	0.000
IR (mm^−1^)	0.004	0.936	−0.625	0.000	−0.328	0.000	−0.511	0.000	0.122	0.004	0.132	0.002	0.110	0.010
SP-A1	0.026	0.547	0.782	0.000	0.558	0.000	0.563	0.000	0.075	0.082	0.046	0.283	−0.229	0.000
CBI	−0.011	0.803	−0.208	0.000	−0.377	0.000	−0.082	0.057	0.105	0.014	−0.032	0.451	−0.035	0.412

*p* < 0.05 considered statistically significant. Rho: Pearson’s correlation coefficient.

Table 6 shows the investigative results of the multivariate linear regression models. Age was negatively associated with HCdArcL and PD, but positively correlated with WEMML. The IOP and CCT were negatively associated with DARM (1 mm), A1V, PD, while being positively related to SP-A1. BIOP was negatively associated with A1V, but the opposite was true when it comes to A2V. ACV was negatively associated with PS, WEMML, and SP-A1.

**TABLE 6:** Results of Stepwise Multiple Regression Analysis between Dynamic Corneal Response Parameters and demographics/ocular characteristics.

Variables	Age(y)^*^			IOP(mmHg)^*^			CCT (μm)^*^			bIOP(mmHg)^*^			Km(D)^*^			Astig(D)^*^			ACV^*^		
	Β	Sta. β	P	β	Sta. β	P	Β	Sta. β	P	Β	Sta. β	P	β	Sta. β	P	Β	Sta. β	P	β	Sta.β	P
A1V (m/s)				−38.532	−0.234	0.000	−582.718	−0.375	0.011	−44.827	−0.306	0.000									
A2V (m/s)										14.961	0.204	0.000									
PD (mm)	−4.179	−0.099	0.028	−2.978	−0.317	0.000	−28.777	−0.299	0.003	−2.608	−0.312	0.000									
Radius (mm)													−0.7.280	−1.521	0.000	−0.209	−0.218	0.000			
HCDLA (mm)													15.556	1.122	0.000						
WEMML (mm)	81.668	0.436	0.000							−8.423	−0.227	0.000				−1.467	−0.141	0.001	−182.982	−0.328	0.002
HCdArcL ([mm)	−76.032	−0.140	0.001																		
DARM [1 mm]				−14.777	−0.264	0.000	−105.792	−0.192	0.032	−9.080	−0.182	0.000									
DARM [2 mm]				1.095	0.124	0.006															
PS (μm)							0.917	0.282	0.005							0.012	0.148	0.000	−2.506	−0.533	0.000
ARTh							0.138	0.301	0.002	−0.004	−0.130	0.000									
IR (mm^−1^)				−0.329	−0.105	0.001															
SP-A1				0.052	0.312	0.000	1.586	0.925	0.000							−0.525	−0.211	0.040	−0.525	−0.211	0.040
CBI																					

Sta.β, Standardized regression coefficient (β); β, regression coefficient (β).

*The coefficients of determination (the adjusted R^2^ value) in multiple linear regressions of age, IOP, CCT, bIOP, Km, Astig, and ACV, were 0.235, 0.782, 0.723, 0.675, 0.493, 0.087, and 0.459, respectively.

The coefficients of determination (the adjusted R^2^ value) in multiple linear regressions of age, IOP, CCT, bIOP, Km, Astig and ACV were 0.235, 0.782, 0.723, 0.675, 0.493, 0.087, and 0.459, respectively.

## Discussion

In recent years, dynamic corneal response (DCR) parameters helped to optimize the interaction between the eye and several treatment and management procedures. The new DCR parameters that were developed with a software upgrade have shown good results in demonstrating biomechanical features in several eye diseases (Tian et al., 2021). A good example is the biomechanical fragility of the keratoconic cornea (Zhang et al., 2021b; Satitpitakul et al., 2021). To the best of our knowledge, a few studies have evaluated the repeatability and reproducibility of the new Corvis ST DCR parameters and bIOP in healthy participants (Matsuura et al., 2019; Serbecic et al., 2020; Wang et al., 2021; Ye et al., 2021). Moreover, the present study was the largest study of corneal biomechanics that explored the association between DCR parameters (provided by Corvis ST incorporating the latest software) and several demographic and ocular characteristics in a Chinese healthy population.

With the new software version in place, the repeatability and reproducibility of the recalculated biomechanical parameters were quite improved. The present study observed that the repeatability and reproducibility of 37 Corvis ST parameters in healthy eyes were good. Kaili Yang et al. found that 46.15% of all the 37 parameters showed excellent repeatability, 25.64% parameters reflected good repeatability, and 28.21% parameters fell under the poor to moderate repeatability range in Chinese healthy eyes. These results were slightly worse than the ones presented in this study, which are 54.05, 37.84, and 8.11%, respectively (Yang et al., 2019). The present study found that A1T, A1V, A2T, A2V, PD, HCDA, A2DA, A1DLL, HCDLL, HCDLA, DLAML, WEMML, WEMMT, HCDLAr, and A1dArcL showed excellent or good repeatability and reproducibility with CV values that are below 20%. Compared with previous studies, we observed a significant decrease of CV values and an increase of ICC values in most DCR parameters (Ali et al., 2014; Wu and Tian, 2016). The results might be due to software upgrade and different population selection. In our study, the CV, CR, and ICC of reproducibility were comparable as far as repeatability was concerned but showed slightly higher values overall. Reproducibility was determined by the random combination of factors such as subject, device, and interactions between the participants and the device (Herber et al., 2020; Serbecic et al., 2020). The controversial results might be due to usage of different devices, varying software versions, and dissimilar population selection.

The results from this study showed that the new parameters had relatively high ICC and low CV values, which was consistent with previous studies to some extent (Vinciguerra et al., 2016a; Roberts et al., 2017). In this study, the new parameters ARTh, IR, SP-A1, CBI, and PS had excellent repeatability and MIR, DARM [1 mm], DARM [2 mm] had good repeatability. The CVs of these parameters were all below 10%. These results were consistent with what Yang and colleagues (Yang et al., 2019) found in that the new parameters presented good repeatability in Chinese healthy eyes. The CV values of CBI were higher than those of other parameters. This might have been caused by the fact that the CBI was a combined parameter that was calculated by a logistic regression analysis, which could result in a large deviation among all the measurements. The bIOP results showed very good sw values for repeatability and reproducibility and this was consistent the findings by Lopesand colleagues(Koprowski et al., 2015). who found that IOP and bIOP presented low CV and sw values in Germany, Italy, and Brazil populations.

Stepwise multiple regression analysis results showed that age increased with larger WEMML, as well as smaller HCdArcL and PD. The WEMML contributed the most for age on the basis of the standardized partial regression coefficient. Several studies reported that the age of the participants exhibited a significant correlation with several corneal biomechanical parameters (Elsheikh et al., 2007; Lee et al., 2018). The studies by Lee et al. and Elsheikh et al. experimentally showed that cornea considerably stiffened with age. An older cornea would probably show lower PD and HCdArcL values. The correlation between WEMML and age might be caused by the changes in the retrobulbar fat composition that occur as years go by. The alterations in retrobulbar fat composition may in turn lead to changes that are associated with ocular displacement under the air puff (Regensburg et al., 2011).

In previous studies, the CCT had crucial influence on the DCR parameters that are provided by the Corvis ST (Daxer et al., 1998). In this study, the CCT was negatively associated with several corneal deformation parameters, such as A1V and DARM [1 mm], thereby suggesting greater deformation during applanation in eyes with thin corneas. In particular, the CCT positively correlated with a new parameter called the SP-A1, which serves as a biomarker for corneal stiffness. The SP-A1 value was reported to be lower in thinner cornea than it is in healthy ones (Vinciguerra et al., 2016a; Zhao et al., 2019). Vinciguerra R and colleagues found that there was a statistically significant decrease in stiffness parameters (SP-A1) and a significant increase in DARM [1 mm] after the PRK and PRK procedure combined with the LASIK procedure. It almost aligns with present results (Vinciguerra et al., 2016b).

In terms of measuring DCR parameters, IOP has a significant impact (Vinciguerra et al., 2016b). In the present study, IOP increased with smaller A1V, DARM [1 mm], IR, as well as larger SP-A1. The PD and SP-A1 contributed the most based on standardized partial regression coefficient, reflecting that corneal stiffness substantially contributes to IOP. Another research study demonstrated that there was a positive linear association between Young’s modulus and IOP by analyzing the stiffness of 37 corneas from human donors (Elsheikh et al., 2008). Moreover, the above results indicate that the cornea is less likely to deform when IOP is high.

The bIOP correction aims to reduce the influence of the cornea’s thickness and age in exhibiting reality IOP values. The bIOP correction has been successfully applied in the estimation of true IOP in *ex vivo* tests that were conducted on human donor eye globes (Eliasy et al., 2018). In the present study, the result of multivariate linear regression models indicated that the PS was more influenced by the CCT but not significantly affected by the bIOP. This finding demonstrated that PS is a good parameter to correctly evaluate *in vivo* corneal biomechanics because of their relative independence from IOP. Furthermore, we found that the bIOP was negatively associated with the A1V, although it was positively associated with A2V. The reason for this may lie in the fact that A2V is not only affected by corneal resistance as it also relates to the viscous damping characteristics of the cornea. The above results might be due to the tissue’s viscous damping property or hysteresis [23].

The anterior chamber may also have some effects on DCR parameters. In this study, the results of multivariable linear model showed that ACV increased with smaller SP-A1, WEMML, and PS. In one study, the researchers found that bigger ACV values were associated with lower HCDA values (Cui et al., 2019). Just like the HCDA, HCDLA eliminates the influence of eye movement. Therefore, bigger ACV values might cause limited eye movement. Furthermore, ACV increased with smaller PS and ARTH. This may suggest that the change of corneal thickness in the central region is larger than it is in the peripheral region. This is due to changes in corneal tension as influenced by high ACV. According to your findings, Nemeth et al., (2017) reported conflicting results compared with ours, with regard to the relationship between ACV and DCR parameters. The explanation to this might be that the participants and parameters for both studies were different. Another possible reason was that ACV could be influenced by a number of factors, such as corneal area, anterior chamber volume, and chamber angle whose association with DCR parameters were not proved. Therefore, further studies are required to expound the association between various factors and the DCR parameters and ACV.

Furthermore, we discovered that Km significantly correlated with PD and HCDLA. These results supported the notion that corneal deformation responses are associated with not only biomechanical properties of the cornea, but the corneal geometric factors, such as Km as well (Fontes et al., 2008; Kamiya et al., 2009).

The coefficient of determination (the R^2^ value) in multiple linear regressions of Astig was 0.087, which is lower than other parameter values. This implies that the variations in DCR parameters explain about 8.7% of the variance of Astig, which implies that Astig was not significantly associated with the biomechanical properties of the eye.

The advantage of this study lies in the large sample size, homogeneous Chinese population origin, and the use of the latest software with new parameters. However, the present study has some limitations. First, as this study only focused on healthy Chinese population, it is not known whether these results can be generalized to other ethnicities, as well as to individuals with other diseases. Second, this study was an observational cross-sectional study that may limit causal inferences.

In conclusion, we profile DCR and bIOP parameters in corneal biomechanical properties as measured by Corvis ST in a large, healthy Chinese population. IOP, CCT, bIOP, Km, and ACV were significantly associated with the DCR parameters of the eye. These results may be relevant for studying the role of altered corneal biomechanics in ocular diseases. As the Corvis ST is a relatively new technology, the applicability and feasibility of this technique in characterizing corneal biomechanics need further investigation.

## Data Availability

The raw data supporting the conclusion of this article will be made available by the authors, without undue reservation.
